# Infectious Disease and Gender^1^

**DOI:** 10.3201/eid1011.040622_05

**Published:** 2004-11

**Authors:** Jeanette H. Magnus, Margaret E. Greene, Sunita Kishor

**Affiliations:** * Tulane School of Public Health and Tropical Medicine, New Orleans, Louisiana, USA;; † Center for Global Health, George Washington University, Washington, DC, USA;; ‡ MEASURE DHS, ORC Macro, Calverton, Maryland, USA

**Keywords:** infectious disease, gender, conference summary

Worldwide, women live longer then men; however, they do not necessarily live better, healthier lives. By the interface of sex and gender, women are placed at high risk for increased illness related to pregnancy and childbirth as well as chronic and infectious diseases.

## Marital Practice

Many young girls around the world are married with household responsibilities and limited access to outside institutions and relationships. More than 30% of 15- to 19-year-old girls in west, east, and central Africa, as well as south and central Asia are married. After marriage, the girls are also less likely to continue education since their husband's family usually does not invest in the education of their daughter-in-law. In South Africa, 45% of 15- to 19-year-old unwed mothers were in school, but only 27% of the wives of the same age were in school. Early childbearing increases maternal death rates substantially, and an estimate of maternal mortality ratio 3 to 7 times higher among 15- to 19-year-old women than among 20- to 34-year-old women is conservative. The timing of marriage, the nature of the relationship, and pressure to marry can place young women at increased risk for disease. In most cultures, the husband is an average of 5–10 years older, and male partners tend to be more sexually experienced. Young women have little negotiating power and are at greater risk of contracting sexually transmitted diseases. Recent studies suggest that young married women are more likely to be HIV positive than their unmarried peers. Cultural demands on the young bride and isolation from peers, friends, and family place her at increased risk for domestic violence and depression.

## Women's Status and Sexually Transmitted Infections

Gender represents the biologic sex of a person and the different roles, rights, and obligations that culture and society attach to persons born with male or female characteristics. Status, power, and rights are attached to gender and affect the health status of women, independent of biologically determined vulnerabilities. Women's risk of acquiring sexually transmitted infections, including HIV/AIDS, is affected by their relative power to want and insist on safe sex, as well as their access to information, condoms, and other resources needed to protect themselves against infection. A 2001–2002 Zambia Demographic and Health Survey found that the odds of having an STI or STI symptom were about twice as high for married Zambian women who have experienced either sexual or physical spousal violence. Also, women who said that getting permission to seek health care was a big hurdle were more likely to have an STI than women who did not have to obtain permission. The study found clear evidence that household-level disempowerment of women is an important risk factor for women having an STI.

